# Hexakis(1-methyl-1*H*-imidazole-κ*N* ^3^)cobalt(II) dibromide dihydrate

**DOI:** 10.1107/S1600536808042906

**Published:** 2009-01-08

**Authors:** Rufu Yao

**Affiliations:** a Department of Chemistry, Hefei Teachers College, Hefei, Anhui 230061, People’s Republic of China

## Abstract

The asymmetric unit of the title compound, [Co(C_4_H_6_N_2_)_6_]Br_2_·2H_2_O, contains one-half of the centrosymmetric cation, one Br atom and one water mol­ecule. The Co^II^ atom, lying on an inversion center, has a distorted octa­hedral geometry, defined by six N atoms from six 1-methyl­imidazole ligands. In the crystal structure, intra- and inter­molecular O—H⋯Br hydrogen bonds link pairs of uncoordinated water mol­ecules and bromide anions.

## Related literature

For general background, see: Lin *et al.* (2007 ▶); Rogers & Seddon (2003 ▶); Xie *et al.* (2008 ▶). For a related structure, see: Baca *et al.* (2005 ▶).
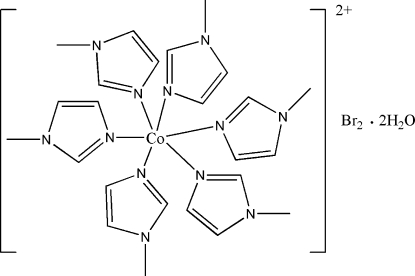


## Experimental

### 

#### Crystal data

[Co(C_4_H_6_N_2_)_6_]Br_2_·2H_2_O
*M* *_r_* = 747.41
Monoclinic, 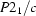

*a* = 8.182 (2) Å
*b* = 13.573 (2) Å
*c* = 16.2340 (19) Å
β = 111.12 (4)°
*V* = 1681.8 (7) Å^3^
*Z* = 2
Mo *K*α radiation
μ = 2.93 mm^−1^
*T* = 298 (2) K
0.40 × 0.30 × 0.30 mm

#### Data collection

Bruker SMART CCD area-detector diffractometer
Absorption correction: multi-scan (*SADABS*; Bruker, 2001 ▶) *T*_min_ = 0.363, *T*_max_ = 0.416
16985 measured reflections
3294 independent reflections
2710 reflections with *I* > 2σ(*I*)
*R*_int_ = 0.071

#### Refinement

*R*[*F* ^2^ > 2σ(*F* ^2^)] = 0.037
*wR*(*F* ^2^) = 0.103
*S* = 1.04
3294 reflections
187 parameters
H-atom parameters constrained
Δρ_max_ = 0.86 e Å^−3^
Δρ_min_ = −0.36 e Å^−3^

### 

Data collection: *SMART* (Bruker, 2001 ▶); cell refinement: *SAINT* (Bruker, 2001 ▶); data reduction: *SAINT*; program(s) used to solve structure: *SHELXS97* (Sheldrick, 2008 ▶); program(s) used to refine structure: *SHELXL97* (Sheldrick, 2008 ▶); molecular graphics: *SHELXTL* (Sheldrick, 2008 ▶); software used to prepare material for publication: *SHELXTL*.

## Supplementary Material

Crystal structure: contains datablocks I, global. DOI: 10.1107/S1600536808042906/hk2594sup1.cif

Structure factors: contains datablocks I. DOI: 10.1107/S1600536808042906/hk2594Isup2.hkl

Additional supplementary materials:  crystallographic information; 3D view; checkCIF report

## Figures and Tables

**Table:** 

Co1—N3	2.174 (2)
Co1—N5	2.182 (2)
Co1—N1	2.207 (2)

**Table:** 

N3^i^—Co1—N3	180.0
N3—Co1—N5	88.07 (8)
N3—Co1—N5^i^	91.93 (8)
N5—Co1—N5^i^	180.0
N3^i^—Co1—N1	87.52 (8)
N3—Co1—N1	92.48 (8)
N5—Co1—N1	89.43 (8)
N5^i^—Co1—N1	90.57 (8)
N1^i^—Co1—N1	180.00 (11)

Symmetry code: (i) 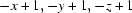.

**Table 2:** Hydrogen-bond geometry (Å, °)

*D*—H⋯*A*	*D*—H	H⋯*A*	*D*⋯*A*	*D*—H⋯*A*
O1*W*—H1*WA*⋯Br1^ii^	0.85	2.57	3.371 (3)	157
O1*W*—H1*WB*⋯Br1	0.86	2.51	3.338 (3)	164

Symmetry code: (ii) 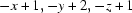.

**Fig. 1.**
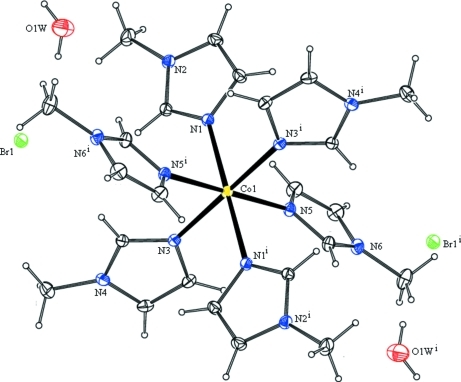
The molecular structure of the title molecule, with the atom-numbering scheme. Displacement ellipsoids are drawn at 30% probability level [symmetry code: (i) 1 - x, 2 - y, 1 - z].

**Fig. 2.**
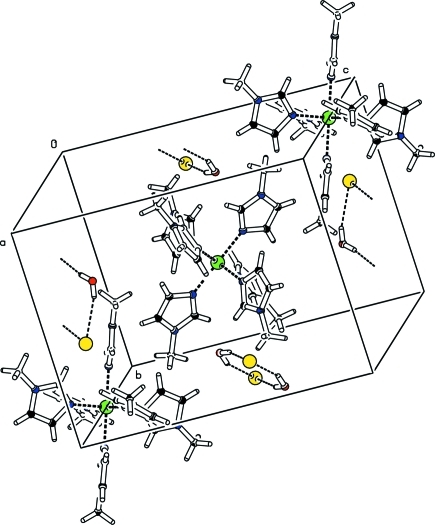
A partial packing diagram of the title compound. Hydrogen bonds are shown as dashed lines.

## supplementary crystallographic information

### Comment

Ionothermal synthesis of novel organic-inorganic hybrid materials are not accessible by traditional hydro- or solvothermal reactions (Rogers & Seddon, 2003, Xie *et al.*, 2008, Lin *et al.*, 2007). We report herein the crystal structure of the title compound.

The asymmetric unit of the title compound (Fig. 1) contains one-half of the centrosymmetric cation, one Br atom and one water molecule. The Co^II^ atom lying on the inversion center of the centrosymmetric cation has a distorted octahedral geometry (Table 1). It is coordinated by six N atoms from six 1-methylimidazole ligands, where N3, N3^i^, N5 and N5^i^ atoms comprise the equatorial plane, and the other two N atoms, N1 and N1^i^, occupy the axial positions [symmetry code: (i) 1 - x, 2 - y, 1 - z]. The Co-N bonds [average value = 2.1877 (2) Å] are longer than the Ni-N bonds [average value = 2.065 Å] in the reported Ni complex with the same ligand (Baca *et al.*, 2005).

In the crystal structure, intra- and intermolecular O-H···Br hydrogen bonds (Table 2) link the pairs of uncoordinated water and bromide anions (Fig. 2), in which they may be effective in the stabilization of the structure.

### Experimental

C_O_(NO_3_)_2_.6H_2_O (0.9 g) and N-methyl imidazole (0.5 g) were placed in a Teflon-line stainless-steel autoclave (25 ml) mixed with 1-ethyl-3-methyl- imidazolium (EMIBr)(1.0 g). The mixtures were heated at 423 K for 3 d, followed by cooling slowly to room temperature. The red block crystals were collected.

### Refinement

H atoms were positioned geometrically, with O-H = 0.8544 and 0.8553 Å (for H_2_O) and C-H = 0.93 and 0.96 Å for aromatic and methyl H, respectively, and constrained to ride on their parent atoms, with U_iso_(H) = xU_eq_(C,O), where x = 1.2 for aromatic H and x = 1.5 for all other H atoms.

### Crystal data

**Table:**

[Co(C_4_H_6_N_2_)_6_]Br_2_·2H_2_O	*F*(000) = 762
*M**_r_* = 747.41	*D*_x_ = 1.47 Mg m^−^^3^
Monoclinic, *P*2_1_/*c*	Mo *K*α radiation, λ = 0.71073 Å
Hall symbol: -P 2ybc	Cell parameters from 7560 reflections
*a* = 8.182 (2) Å	θ = 2.5–27.1°
*b* = 13.573 (2) Å	µ = 2.93 mm^−^^1^
*c* = 16.2340 (19) Å	*T* = 298 K
β = 111.12 (4)°	Block, red
*V* = 1681.8 (7) Å^3^	0.40 × 0.30 × 0.30 mm
*Z* = 2	

### Data collection

**Table:**

Bruker SMART CCD area-detector diffractometer	3294 independent reflections
Radiation source: fine-focus sealed tube	2710 reflections with *I* > 2σ(*I*)
graphite	*R*_int_ = 0.071
φ and ω scans	θ_max_ = 26.0°, θ_min_ = 2.0°
Absorption correction: multi-scan (*SADABS*; Bruker, 2001)	*h* = −9→10
*T*_min_ = 0.363, *T*_max_ = 0.416	*k* = −15→16
16985 measured reflections	*l* = −20→20

### Refinement

**Table:**

Refinement on *F*^2^	Primary atom site location: structure-invariant direct methods
Least-squares matrix: full	Secondary atom site location: difference Fourier map
*R*[*F*^2^ > 2σ(*F*^2^)] = 0.037	Hydrogen site location: inferred from neighbouring sites
*wR*(*F*^2^) = 0.103	H-atom parameters constrained
*S* = 1.03	*w* = 1/[σ^2^(*F*_o_^2^) + (0.0649*P*)^2^ + 0.2015*P*] where *P* = (*F*_o_^2^ + 2*F*_c_^2^)/3
3294 reflections	(Δ/σ)_max_ = 0.001
187 parameters	Δρ_max_ = 0.86 e Å^−^^3^
0 restraints	Δρ_min_ = −0.36 e Å^−^^3^

### Special details

**Table:**

Geometry. All e.s.d.'s (except the e.s.d. in the dihedral angle between two l.s. planes) are estimated using the full covariance matrix. The cell e.s.d.'s are taken into account individually in the estimation of e.s.d.'s in distances, angles and torsion angles; correlations between e.s.d.'s in cell parameters are only used when they are defined by crystal symmetry. An approximate (isotropic) treatment of cell e.s.d.'s is used for estimating e.s.d.'s involving l.s. planes.
Refinement. Refinement of *F*^2^ against ALL reflections. The weighted *R*-factor *wR* and goodness of fit *S* are based on *F*^2^, conventional *R*-factors *R* are based on *F*, with *F* set to zero for negative *F*^2^. The threshold expression of *F*^2^ > σ(*F*^2^) is used only for calculating *R*-factors(gt) *etc*. and is not relevant to the choice of reflections for refinement. *R*-factors based on *F*^2^ are statistically about twice as large as those based on *F*, and *R*- factors based on ALL data will be even larger.

### Fractional atomic coordinates and isotropic or equivalent isotropic displacement parameters (Å^2^)

**Table:**

	*x*	*y*	*z*	*U*_iso_*/*U*_eq_	
Co1	0.5000	0.5000	0.5000	0.03601 (15)	
Br1	0.78110 (4)	0.87845 (2)	0.56859 (2)	0.05972 (14)	
O1W	0.4155 (4)	0.9171 (2)	0.3940 (2)	0.1105 (11)	
H1WA	0.3888	0.9772	0.3983	0.166*	
H1WB	0.4947	0.9012	0.4433	0.166*	
N1	0.3436 (3)	0.62856 (14)	0.51072 (14)	0.0415 (5)	
N2	0.2626 (3)	0.77351 (16)	0.54359 (16)	0.0490 (5)	
N3	0.7232 (3)	0.54470 (15)	0.61490 (13)	0.0419 (5)	
N4	0.9664 (3)	0.61811 (16)	0.70086 (15)	0.0504 (6)	
N5	0.4033 (3)	0.41844 (16)	0.58900 (13)	0.0433 (5)	
N6	0.3497 (3)	0.30035 (18)	0.66980 (15)	0.0539 (6)	
C1	0.3966 (3)	0.71343 (19)	0.54940 (17)	0.0449 (6)	
H1A	0.5138	0.7302	0.5777	0.054*	
C2	0.1629 (4)	0.6342 (2)	0.4783 (2)	0.0543 (7)	
H2A	0.0873	0.5845	0.4474	0.065*	
C3	0.1130 (4)	0.7236 (2)	0.4986 (2)	0.0567 (7)	
H3A	−0.0012	0.7463	0.4845	0.068*	
C4	0.2759 (5)	0.8748 (2)	0.5786 (3)	0.0792 (12)	
H4A	0.3971	0.8920	0.6075	0.119*	
H4B	0.2173	0.8785	0.6202	0.119*	
H4C	0.2220	0.9198	0.5309	0.119*	
C5	0.8328 (3)	0.61851 (19)	0.62204 (17)	0.0444 (6)	
H5A	0.8195	0.6651	0.5781	0.053*	
C6	0.7915 (4)	0.4943 (2)	0.69380 (18)	0.0521 (7)	
H6A	0.7419	0.4386	0.7085	0.063*	
C7	0.9419 (4)	0.5382 (2)	0.74685 (19)	0.0564 (7)	
H7A	1.0137	0.5181	0.8030	0.068*	
C8	1.1066 (5)	0.6909 (3)	0.7314 (3)	0.0799 (10)	
H8A	1.0926	0.7386	0.6857	0.120*	
H8B	1.2178	0.6586	0.7457	0.120*	
H8C	1.1017	0.7234	0.7830	0.120*	
C9	0.4120 (4)	0.3232 (2)	0.60596 (17)	0.0480 (6)	
H9A	0.4561	0.2770	0.5771	0.058*	
C10	0.3301 (4)	0.4577 (2)	0.6465 (2)	0.0626 (8)	
H10A	0.3063	0.5242	0.6502	0.075*	
C11	0.2980 (5)	0.3862 (2)	0.6964 (2)	0.0663 (9)	
H11A	0.2502	0.3940	0.7400	0.080*	
C12	0.3336 (6)	0.2008 (3)	0.7011 (2)	0.0862 (12)	
H12A	0.3805	0.1539	0.6713	0.129*	
H12B	0.2123	0.1863	0.6890	0.129*	
H12C	0.3973	0.1970	0.7636	0.129*	

### Atomic displacement parameters (Å^2^)

**Table:**

	*U*^11^	*U*^22^	*U*^33^	*U*^12^	*U*^13^	*U*^23^
Co1	0.0387 (3)	0.0319 (3)	0.0416 (2)	0.00008 (18)	0.0197 (2)	0.00050 (18)
Br1	0.0506 (2)	0.0458 (2)	0.0787 (2)	−0.00077 (12)	0.01838 (17)	−0.01020 (13)
O1W	0.099 (2)	0.096 (2)	0.109 (2)	0.0179 (17)	0.0042 (18)	−0.0335 (18)
N1	0.0405 (12)	0.0390 (12)	0.0502 (11)	0.0004 (9)	0.0227 (10)	−0.0009 (9)
N2	0.0508 (14)	0.0370 (12)	0.0683 (13)	0.0033 (9)	0.0325 (11)	−0.0024 (10)
N3	0.0450 (12)	0.0370 (11)	0.0444 (11)	0.0026 (9)	0.0168 (9)	0.0005 (9)
N4	0.0388 (12)	0.0544 (14)	0.0536 (13)	0.0033 (10)	0.0112 (11)	0.0010 (10)
N5	0.0483 (13)	0.0421 (12)	0.0465 (11)	−0.0038 (10)	0.0255 (10)	−0.0006 (9)
N6	0.0603 (15)	0.0592 (15)	0.0490 (12)	−0.0095 (12)	0.0278 (11)	0.0090 (11)
C1	0.0404 (14)	0.0441 (14)	0.0560 (14)	0.0005 (11)	0.0244 (12)	−0.0027 (12)
C2	0.0385 (15)	0.0564 (17)	0.0677 (17)	0.0003 (12)	0.0185 (13)	−0.0103 (14)
C3	0.0411 (15)	0.0606 (18)	0.0697 (17)	0.0099 (13)	0.0217 (13)	−0.0048 (14)
C4	0.083 (3)	0.0403 (18)	0.130 (3)	0.0009 (15)	0.057 (3)	−0.0167 (17)
C5	0.0403 (14)	0.0454 (15)	0.0463 (14)	0.0014 (11)	0.0143 (12)	0.0047 (11)
C6	0.0621 (18)	0.0408 (15)	0.0522 (15)	0.0038 (13)	0.0191 (14)	0.0072 (12)
C7	0.0588 (19)	0.0551 (17)	0.0480 (15)	0.0161 (14)	0.0104 (14)	0.0064 (13)
C8	0.056 (2)	0.083 (3)	0.082 (2)	−0.0173 (18)	0.0011 (17)	−0.0009 (19)
C9	0.0593 (17)	0.0474 (16)	0.0450 (13)	−0.0076 (12)	0.0284 (12)	0.0002 (11)
C10	0.076 (2)	0.0598 (18)	0.0685 (18)	0.0081 (16)	0.0465 (17)	0.0004 (15)
C11	0.074 (2)	0.080 (2)	0.0643 (18)	0.0034 (17)	0.0493 (17)	0.0025 (16)
C12	0.121 (3)	0.071 (2)	0.082 (2)	−0.017 (2)	0.056 (2)	0.0216 (19)

### Geometric parameters (Å, °)

**Table:**

Co1—N3^i^	2.174 (2)	N6—C12	1.466 (4)
Co1—N3	2.174 (2)	C1—H1A	0.9300
Co1—N5	2.182 (2)	C2—C3	1.359 (4)
Co1—N5^i^	2.182 (2)	C2—H2A	0.9300
Co1—N1^i^	2.207 (2)	C3—H3A	0.9300
Co1—N1	2.207 (2)	C4—H4A	0.9600
O1W—H1WA	0.8544	C4—H4B	0.9600
O1W—H1WB	0.8553	C4—H4C	0.9600
N1—C1	1.309 (3)	C5—H5A	0.9300
N1—C2	1.381 (4)	C6—C7	1.359 (4)
N2—C1	1.342 (3)	C6—H6A	0.9300
N2—C3	1.359 (4)	C7—H7A	0.9300
N2—C4	1.477 (4)	C8—H8A	0.9600
N3—C5	1.322 (3)	C8—H8B	0.9600
N3—C6	1.380 (3)	C8—H8C	0.9600
N4—C5	1.351 (3)	C9—H9A	0.9300
N4—C7	1.372 (4)	C10—C11	1.349 (4)
N4—C8	1.458 (4)	C10—H10A	0.9300
N5—C9	1.319 (4)	C11—H11A	0.9300
N5—C10	1.384 (3)	C12—H12A	0.9600
N6—C9	1.346 (3)	C12—H12B	0.9600
N6—C11	1.362 (4)	C12—H12C	0.9600
			
N3^i^—Co1—N3	180.0	N2—C3—C2	106.5 (2)
N3^i^—Co1—N5	91.93 (8)	N2—C3—H3A	126.8
N3—Co1—N5	88.07 (8)	C2—C3—H3A	126.8
N3^i^—Co1—N5^i^	88.07 (8)	N2—C4—H4A	109.5
N3—Co1—N5^i^	91.93 (8)	N2—C4—H4B	109.5
N5—Co1—N5^i^	180.0	H4A—C4—H4B	109.5
N3^i^—Co1—N1^i^	92.48 (8)	N2—C4—H4C	109.5
N3—Co1—N1^i^	87.52 (8)	H4A—C4—H4C	109.5
N5—Co1—N1^i^	90.57 (8)	H4B—C4—H4C	109.5
N5^i^—Co1—N1^i^	89.43 (8)	N3—C5—N4	111.9 (2)
N3^i^—Co1—N1	87.52 (8)	N3—C5—H5A	124.1
N3—Co1—N1	92.48 (8)	N4—C5—H5A	124.1
N5—Co1—N1	89.43 (8)	C7—C6—N3	110.1 (3)
N5^i^—Co1—N1	90.57 (8)	C7—C6—H6A	125.0
N1^i^—Co1—N1	180.00 (11)	N3—C6—H6A	125.0
H1WA—O1W—H1WB	107.2	C6—C7—N4	106.2 (2)
C1—N1—C2	105.0 (2)	C6—C7—H7A	126.9
C1—N1—Co1	129.22 (18)	N4—C7—H7A	126.9
C2—N1—Co1	125.80 (17)	N4—C8—H8A	109.5
C1—N2—C3	106.9 (2)	N4—C8—H8B	109.5
C1—N2—C4	126.4 (2)	H8A—C8—H8B	109.5
C3—N2—C4	126.7 (2)	N4—C8—H8C	109.5
C5—N3—C6	105.0 (2)	H8A—C8—H8C	109.5
C5—N3—Co1	128.35 (17)	H8B—C8—H8C	109.5
C6—N3—Co1	126.31 (18)	N5—C9—N6	112.3 (2)
C5—N4—C7	106.9 (2)	N5—C9—H9A	123.8
C5—N4—C8	126.1 (3)	N6—C9—H9A	123.8
C7—N4—C8	127.0 (3)	C11—C10—N5	110.7 (3)
C9—N5—C10	103.9 (2)	C11—C10—H10A	124.7
C9—N5—Co1	129.10 (17)	N5—C10—H10A	124.7
C10—N5—Co1	126.8 (2)	C10—C11—N6	106.0 (2)
C9—N6—C11	107.1 (2)	C10—C11—H11A	127.0
C9—N6—C12	125.8 (3)	N6—C11—H11A	127.0
C11—N6—C12	127.0 (2)	N6—C12—H12A	109.5
N1—C1—N2	112.3 (2)	N6—C12—H12B	109.5
N1—C1—H1A	123.8	H12A—C12—H12B	109.5
N2—C1—H1A	123.8	N6—C12—H12C	109.5
C3—C2—N1	109.4 (3)	H12A—C12—H12C	109.5
C3—C2—H2A	125.3	H12B—C12—H12C	109.5
N1—C2—H2A	125.3		
			
N3^i^—Co1—N1—C1	−157.5 (2)	C3—N2—C1—N1	0.5 (3)
N3—Co1—N1—C1	22.5 (2)	C4—N2—C1—N1	−178.9 (3)
N5—Co1—N1—C1	110.5 (2)	C1—N1—C2—C3	0.3 (3)
N5^i^—Co1—N1—C1	−69.5 (2)	Co1—N1—C2—C3	178.83 (19)
N3^i^—Co1—N1—C2	24.3 (2)	C1—N2—C3—C2	−0.3 (3)
N3—Co1—N1—C2	−155.7 (2)	C4—N2—C3—C2	179.1 (3)
N5—Co1—N1—C2	−67.7 (2)	N1—C2—C3—N2	0.0 (3)
N5^i^—Co1—N1—C2	112.3 (2)	C6—N3—C5—N4	−0.1 (3)
N5—Co1—N3—C5	−161.6 (2)	Co1—N3—C5—N4	−173.74 (17)
N5^i^—Co1—N3—C5	18.4 (2)	C7—N4—C5—N3	0.7 (3)
N1^i^—Co1—N3—C5	107.7 (2)	C8—N4—C5—N3	−177.7 (3)
N1—Co1—N3—C5	−72.3 (2)	C5—N3—C6—C7	−0.5 (3)
N5—Co1—N3—C6	26.0 (2)	Co1—N3—C6—C7	173.29 (18)
N5^i^—Co1—N3—C6	−154.0 (2)	N3—C6—C7—N4	0.9 (3)
N1^i^—Co1—N3—C6	−64.6 (2)	C5—N4—C7—C6	−0.9 (3)
N1—Co1—N3—C6	115.4 (2)	C8—N4—C7—C6	177.4 (3)
N3^i^—Co1—N5—C9	78.8 (2)	C10—N5—C9—N6	−0.1 (3)
N3—Co1—N5—C9	−101.2 (2)	Co1—N5—C9—N6	175.28 (18)
N1^i^—Co1—N5—C9	−13.7 (2)	C11—N6—C9—N5	−0.3 (3)
N1—Co1—N5—C9	166.3 (2)	C12—N6—C9—N5	177.0 (3)
N3^i^—Co1—N5—C10	−106.8 (2)	C9—N5—C10—C11	0.4 (4)
N3—Co1—N5—C10	73.2 (2)	Co1—N5—C10—C11	−175.1 (2)
N1^i^—Co1—N5—C10	160.7 (2)	N5—C10—C11—N6	−0.6 (4)
N1—Co1—N5—C10	−19.3 (2)	C9—N6—C11—C10	0.5 (4)
C2—N1—C1—N2	−0.4 (3)	C12—N6—C11—C10	−176.8 (3)
Co1—N1—C1—N2	−178.95 (16)		

Symmetry codes: (i) −*x*+1, −*y*+1, −*z*+1.

### Hydrogen-bond geometry (Å, °)

**Table:**

*D*—H···*A*	*D*—H	H···*A*	*D*···*A*	*D*—H···*A*
O1W—H1WA···Br1^ii^	0.85	2.57	3.371 (3)	157
O1W—H1WB···Br1	0.86	2.51	3.338 (3)	164

Symmetry codes: (ii) −*x*+1, −*y*+2, −*z*+1.
